# Supplementary material for: Prostaglandin E2 (PGE2) and Roflumilast Involvement in IPF Progression
Source: Int J Mol Sci. 2023 Aug 3;24(15):12393. doi: 10.3390/ijms241512393 (PMC10418473; doi:10.3390/ijms241512393)
Supplement: Supplementary file 1 [file ijms-24-12393-s001.zip › ijms-2504418-supplementary.pdf]

Supplementary Table S1: Antibodies used in the study

**Target**	**Cat. No**	**Source**	**Dilution**	**Company**
HIF1α	Ab2185	Rabbit	1:1000 (WB)	Abcam
SMA	#19245	Rabbit	1:1000 (WB)	Cell Signaling Technologies
PAI-1	Ab66705	Rabbit	1:2000 (WB)	Abcam
Beta-Actin	#8457	Rabbit mAb	1:1000 (WB)	Cell Signaling Technologies
Peroxidase conjugated anti-mouse	#AP308P	Goat	1:5000 (WB)	Millipore
Peroxidase conjugated anti-Rabbit	#AP132P	Goat	1:5000 (WB)	Millipore
